# EUCANEXT: an integrated database for the exploration of genomic and transcriptomic data from *Eucalyptus* species

**DOI:** 10.1093/database/bax079

**Published:** 2017-10-24

**Authors:** Leandro Costa Nascimento, Marcela Mendes Salazar, Jorge Lepikson-Neto, Eduardo Leal Oliveira Camargo, Lucas Salera Parreiras, Gonçalo Amarante Guimarães Pereira, Marcelo Falsarella Carazzolle

**Affiliations:** 1 Laboratório de Genômica e Expressão (LGE), Departamento de Genética, Evolução e Bioagentes, Instituto de Biologia, Universidade Estadual de Campinas, Campinas, SP, Brasil; 2 Laboratório Central de Tecnologias de Alto Desempenho (LaCTAD), Universidade Estadual de Campinas, Campinas, SP, Brasil; 3 Centro Nacional de Processamento de Alto Desempenho em São Paulo (CENAPAD), Universidade Estadual de Campinas, Campinas, SP, Brasil

## Abstract

Tree species of the genus *Eucalyptus* are the most valuable and widely planted hardwoods in the world. Given the economic importance of *Eucalyptus* trees, much effort has been made towards the generation of specimens with superior forestry properties that can deliver high-quality feedstocks, customized to the industrýs needs for both cellulosic (paper) and lignocellulosic biomass production. In line with these efforts, large sets of molecular data have been generated by several scientific groups, providing invaluable information that can be applied in the development of improved specimens. In order to fully explore the potential of available datasets, the development of a public database that provides integrated access to genomic and transcriptomic data from *Eucalyptus* is needed. EUCANEXT is a database that analyses and integrates publicly available *Eucalyptus* molecular data, such as the *E. grandis* genome assembly and predicted genes, ESTs from several species and digital gene expression from 26 RNA-Seq libraries. The database has been implemented in a Fedora Linux machine running MySQL and Apache, while Perl CGI was used for the web interfaces. EUCANEXT provides a user-friendly web interface for easy access and analysis of publicly available molecular data from *Eucalyptus* species. This integrated database allows for complex searches by gene name, keyword or sequence similarity and is publicly accessible at http://www.lge.ibi.unicamp.br/eucalyptusdb. Through EUCANEXT, users can perform complex analysis to identify genes related traits of interest using RNA-Seq libraries and tools for differential expression analysis. Moreover, all the bioinformatics pipeline here described, including the database schema and PERL scripts, are readily available and can be applied to any genomic and transcriptomic project, regardless of the organism.

**Database URL:** http://www.lge.ibi.unicamp.br/eucalyptusdb

## Introduction

The *Eucalyptus* genus is composed by more than 700 species and includes the most extensively planted hardwood trees in the world (1, 2). Currently, about 20 of these species are used in commercial plantations in over 100 countries, being employed mainly for timber, pulp and paper production (3). In addition, as the lignocellulosic biofuels industry advances, *Eucalyptus* trees may become an important feedstock for the production of renewable fuels (4), such as cellulosic ethanol (5, 6). The economic importance of these trees drives intense efforts in the development of specimens with industrially desirable properties, such as increased productivity and lower production costs. Traditionally, the development of trees with desired properties has been achieved through long-lasting breeding programs. More recently, genomic and transcriptomic studies have emerged as a promising tool to be applied in genome-assisted breeding programs and in the development of transgenic technologies.

Starting in the year of 2000, a number of *Eucalyptus* genomic studies have been published, including some that were produced by international initiatives. These studies produced a wide range of molecular data, including ESTs (7–9), microarrays (10–12) and SAGE (13). More recently, the genome sequencing of the *Eucalyptus* species *E. grandis* (14), *E. camaldulensis* (draft genome) (15) and *E. globulus* (in progress) (16) representing a great achievement, potentially turning *Eucalyptus* into a model genus for studies of woody plants. Advancements in sequencing technologies and bioinformatics software have resulted in lower costs for a number of next-generation sequencing (NGS) applications, including RNA-sequencing (RNA-Seq) (17). Consequently, a number of studies have explored the *Eucalyptus* transcriptome through RNA-Seq. This has been analysed by different methodologies considering either mapping the reads into the genes (18) or performing a *de novo* (without a reference) assembly of reads (19–21). Each RNA-Seq library produces massive amounts of data, creating the need for the development of new pipelines for processing and analysing these data in a standardized way. Web-based databases which integrate genomic and transcriptomic data from several tissues and conditions are a valuable tool to facilitate the mining of genes, promoters and expression profiles of biotechnological interest. In the literature, there are few available *Eucalyptus* databases: Eucatoul (http://www.polebio.lrsv.ups-tlse.fr/eucatoul), which stores two ESTs datasets related to wood formation (22) and cold tolerance (23); *Eucalyptus camaldulensis* Genome Database (http://www.kazusa.or.jp/eucaly/), which keeps the draft genomic data (genome and gene prediction) of *E. camaldulensis* (15); and an expressed gene catalogue of the hybrid species *Eucalyptus urophylla* versus *E. grandis* (called Eucspresso—http://eucspresso.bi.up.ac.za) (19). Despite the usefulness of the available *Eucalyptus* databases, there is still the need for a database that integrates all the data available from the multiple transcriptome experiments together with published *Eucalyptus* genome assemblies. There are several integrated databases available online, like PopGenIE (The Populus Genome Integrative Explorer) (24), Phytozome (25) and EucGeniE (The Eucalyptus Genome Integrative Explorer—https://eucgenie.org/) (26), but none of them supports data from *Eucalyptus* genome integrated with all public transcriptome data, allow the user to submit new RNA-Seq data and perform differential expression analysis.

Addressing this issue, we developed a new database, called EUCANEXT, with web-based tools for the exploration of the available *Eucalyptus* transcriptome datasets. The database integrates digital gene expression (provided by RNA-Seq) with the published *E. grandis* genome and ESTs data from several *Eucalyptus* species and experimental conditions. The web interface (http://www.lge.ibi.unicamp.br/eucalyptusdb) allows users to search by keyword, sequence similarity (local BLAST) or transcript ID. Moreover, EUCANEXT allows the user to identify a set of differentially expressed genes in different conditions selected in the web interface. Finally, all scripts used to build the EUCANEXT database, the source codes of the web interfaces and the SQL *schema* are readily available for download on the website, allowing the method described here to be applied to any genomic and transcriptomic project, regardless of the organism.

## Database content

### Genomic data

The *Eucalyptus grandis* genome v 2.0 (11 chromosomes and 4,932 scaffolds), predicted genes (36,349 loci and 46,280 transcripts) and GFF file containing the positions of the transcripts in the reference genome were downloaded from Phytozome (25) v 10.2 (http://phytozome.jgi.doe.gov/pz/portal.html).

### ESTs data

**Figure 1.**
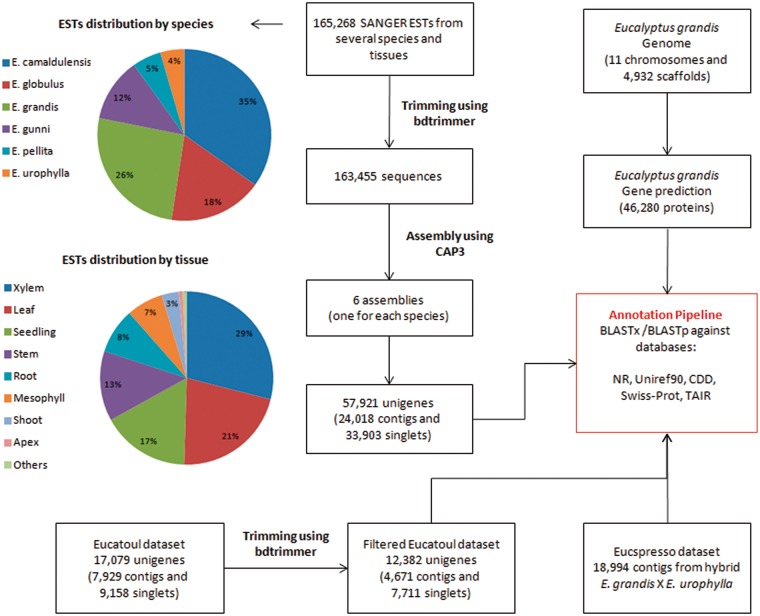
All assembled sequences (from six ESTs assemblies, Eucatoul and Eucspresso), and the *E. grandis* transcripts were automatically annotated with BLAST against NR, Swiss-prot, Uniref90, TAIR and CDD.

A total of 165,268 ESTs sequences (sequenced by SANGER technology) from several *Eucalyptus* species and tissues were obtained from NCBI (Table 1, Figure 1 and Supplementary File S1). The *bdtrimmer* software version 1.1 with default parameters (27) was used to exclude ribosomal, vector, low-quality and short (<100 bp) sequences. Six individual assemblies (one for each species) were performed using the CAP3 program (28) with default parameters.

**Table 1.** Summary of ESTs sequences of EUCANEXT database

Species	Sequences	Unigenes	Annotated unigenes (Blastx—NR)
*E. camaldulensis*	57,602	16,669	13,194
*E. globulus*	28,949	10,724	9,360
*E. grandis*	42,575	15,243	13,242
*E. gunni*	19,841	6,878	5,060
*E. pellita*	8,870	4,360	3,856
*E. urophylla*	7,431	4,047	3,435

### Eucspresso data

A public catalog containing expressed genes of the commercially available hybrid *E. grandis* × *E. urophylla* (‘*E. Urograndis*’) was downloaded from the Eucspresso website. The data set consists of 18,994 contigs >200 bp with 99.8% of the contigs having sequence similarity to the *E. grandis* genome assembly (19).

### Eucatool data

A total of 17,079 unigenes (7,929 contigs and 9,158 singlets) were downloaded from Eucatoul (http://www.polebio.lrsv.ups-tlse.fr/eucatoul) (22, 23). This dataset was filtered using the *bdtrimmer* software version 1.1 (27) to exclude poly-A/T regions and short sequences (<100 bp). The final Eucatoul dataset is composed of 12,382 unigenes, with 4,671 contigs and 7,711 singlets (Figure 1).

### RNA-Seq data

A total of 14 Illumina RNA-Seq libraries from *Eucalyptus* xylem tissues were used: 1 of *Eucalyptus grandis*, 1 of *Eucalyptus globulus*, 1 of *Eucalyptus urophylla* (20) and 11 of a commercial hybrid *Eucalyptus grandis* × *Eucalyptus urophylla* subjected to different experimental conditions (18, 21). In addition, 12 Illumina RNA-Seq libraries from *Eucalyptus camaldulensis* seedlings were downloaded from the SRA (http://www.ncbi.nlm.nih.gov/sra) (29) (Table 2). All reads were aligned against the *Eucalyptus grandis* transcripts using the RSEM aligner (30) version 1.2.19 with default parameters. The RSEM returns gene expression of each transcript using the RPKM (Reads Per Kilobase of exon per Million fragments mapped) or FPKM (Fragments Per Kilobase of transcripts per Million mapped reads) values (31). In addition, the reads were aligned against the *Eucalyptus grandis* genome using TopHat aligner (32) version 2.0.14 with minimum intron size 10 (-i 10) (Table 2).

**Table 2.** Summary of Illumina RNA-Seq libraries available at EUCANEXT database

Species	Condition	Reads	Mapped reads (genome)	SRA accession
*E. camaldulensis*	Leaf collected in the dry tropics (KC0)	3,324,731	2,751,229	SRR521589
*E. camaldulensis*	Leaf submitted to the water stress collected in the dry tropics (KC1)	2,592,563	2,081,961	SRR521590
*E. camaldulensis*	Leaf from seedlings collected in the dry tropics (KS0)	4,176,732	3,258,567	SRR521591
*E. camaldulensis*	Leaf from seedlings submitted to the water stress collected in the dry tropics (KS1)	6,700,497	5,458,101	SRR521592
*E. camaldulensis*	Leaf collected in the semi-arid tropics (MC0)	4,557,996	3,462,320	SRR521593
*E. camaldulensis*	Leaf submitted to the water stress collected in the semi-arid tropics (MC1)	2,501,584	1,914,332	SRR521594
*E. camaldulensis*	Leaf from seedlings collected in the semi-arid tropics (MS0)	2,209,441	1,836,002	SRR521595
*E. camaldulensis*	Leaf from seedlings submitted to the water stress collected in the semi-arid tropics (MS1)	3,044,862	2,470,465	SRR521596
*E. camaldulensis*	Leaf collected in the humid (PC0)	2,423,663	2,030,890	SRR521597
*E. camaldulensis*	Leaf submitted to the water stress collected in the humid tropics (PC1)	3,905,442	3,180,781	SRR521598
*E. camaldulensis*	Leaf from seedlings collected in the humid tropics (PS0)	8,056,075	6,704,333	SRR521599
*E. camaldulensis*	Leaf from seedlings submitted to the water stress collected in the humid tropics (PS1)	9,038,516	7,478,539	SRR521600
*E. grandis*	Xylem	24,679,724	23,025,083	SRR2602746
*E. globulus*	Xylem	28,838,976	26,542,858	SRR2602747
*E. urophylla*	Xylem	25,207,059	23,414,707	SRR2602748
*E. urograndis*	Xylem control; replicate 1	32,076,198	28,361,530	SRR1598974
*E. urograndis*	Xylem narigenin-chalcone supplemented 5 months	34,157,958	31,220,393	SRR1598984
*E. urograndis*	Xylem narigenin supplemented 5 months, replicate 1	33,743,449	30,824,831	SRR1598985
*E. urograndis*	Xylem control; replicate 2	47,260,461	43,159,632	SRR1598989
*E. urograndis*	Xylem narigenin supplemented 5 months, replicate 2	43,768,249	39,530,378	SRR1598990
*E. urograndis*	Xylem narigenin-chalcone supplemented 30 days	54,985,740	50,045,810	SRR1598991
*E. urograndis*	Xylem narigenin supplemented 30 days	46,415,197	42,119,735	SRR1598992
*E. urograndis*	Xylem limiting N fertilization	30,226,072	27,442,383	SRR1561161
*E. urograndis*	Xylem regular N fertilization	30,761,294	28,568,094	SRR1561153
*E. urograndis*	Xylem luxuriant N fertilization (NH4+treatment)	27,558,333	25,711,217	SRR1561174
*E. urograndis*	Xylem NO3 fertilization	27,814,531	25,968,057	SRR1561176

### Gene annotation

The *Eucalyptus* transcripts and the assembled unigenes (assembled ESTs, Eucspresso and Eucatool sequences) were submited to an automatic annotation pipeline based on sequence comparison using BLASTp/x (e-value cutoff of 1e^−5^) against several protein databases, which included: non-redundant (NR) database of *NCBI*, uniref90—database containing clustered sets of proteins from UniProt (33), CDD—a database of conserved domains (34), Swiss-prot—database manually annotated and reviewed from Uniprot (35) and TAIR (The Arabidopsis Information Resource) version 10 (36). In addition, it was included the *E. grandis* transcript annotation generated by Phytozome platform. The Gene Onotlogy annotation (GO) was also extracted from Phytozome data.

### Orthologs identification

The software OrthoMCL (37) with default parameters was used in order to identify orthologs of the *Eucalyptus grandis* loci in three species: *Arabidopsis thaliana* (TAIR 10, 27,416 gene loci) (36), *Populus tricocarpha* (v. 3.0, 41,335 gene loci) (38) and *Glycine max* (v Wm82.a2.v1, 56,044 gene loci) (39). The program ran separately for each species and the output file containing the best directional hit between the species was used for ortholog inference.

### Co-expression analysis

The WGCNA package (40) was used to construct the gene coexpression network. The log2 of normalized gene expression matrix formed by all RNA-Seq libraries available at EUCANEXT was transformed into adjacency matrix using signed Pearson correlation and a soft-threshold power of 12 (chosen by scale-free topology criterion). A correlation threshold of 0.78 was chosen to cut the dendogram into distinct modules. The coexpressed genes pairs for each module were generated using a Pearson correlation of 0.9.

### Database implementation

**Figure 2.**
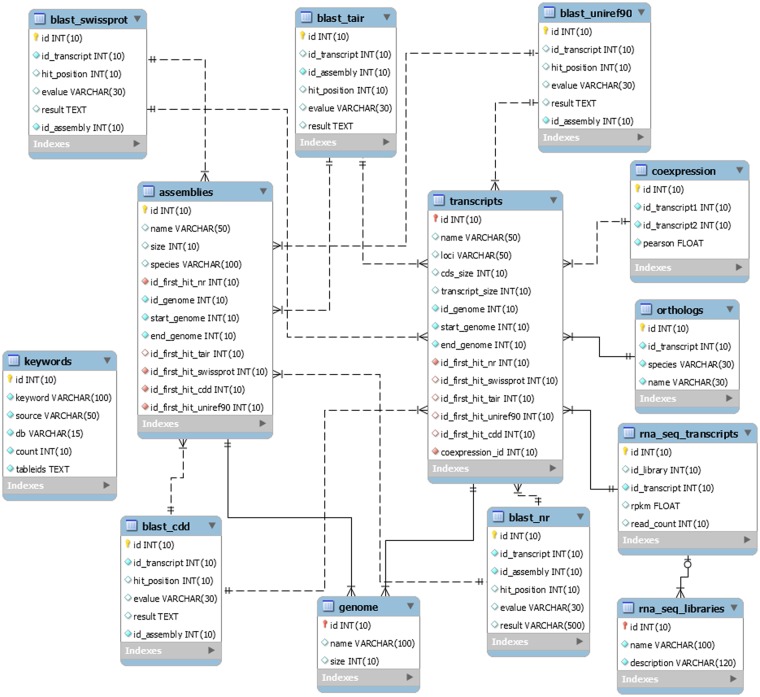
The schema was generated by the MySQL Workbench 5.2 (http://www.mysql.com/products/workbench) and not contains the tables downloaded from the Gene Ontology Consortium.

The EUCANEXT is hosted in a Fedora Linux machine, running the MySQL database server. The web interfaces (available at http://www.lge.ibi.unicamp.br/eucalyptusdb) are based on a combination of CGI scripts using PERL language (including BioPerl module) and the Apache Web Server. The complete schema of the database (Figure 2) is developed to integrate all data and facilitate the web access. The schema is subdivided into four sections, (i) genomic, (ii) ESTs, (iii) RNA-Seq and (iv) annotation, all of which are further described below.

### Genomic section

The genomic section is the core of the EUCANEXT database. The table named ‘genome’ stores the information about the *Eucalyptus grandis* genome assembly, including sequences name and size. The table named ‘transcripts’ keeps the information about the *Eucalyptus grandis* prediction, including sequences name, CDS and transcript sizes, locus and annotation (see the annotation section). The tables ‘genome’ and ‘transcripts’ are linked by the position of the transcripts in the reference genome (fields ‘id_genome’, ‘start_genome’ and ‘end_genome’).

### ESTs section

The ESTs section keeps all the information about ESTs assemblies. In the table ‘ests’, the sequences names are the same ones from NCBI. It is also possible to obtain information about the sequences size (raw and trimmed), species and annotation (see the annotation section). This table is linked to the genomic section by the positions of the ESTs in the genome, obtained by the Exonerate aligner—see Gene browser configuration (fields ‘id_genome’, ‘start_genome’ and ‘end_genome’). The information about the assemblies can be retrieved from the tables ‘assemblies’ and ‘assemblies_ests’. The ‘assemblies’ table contains information about the unigenes, including name, size and annotation (see the annotation section). The table ‘assemblies_ests’ is responsible for crossing the information of the tables ‘ests’ and ‘assemblies’ (fields ‘id_est’, ‘id_assembly’).

### RNA-Seq section

The RNA-Seq section is formed by two tables: ‘rna_seq_libraries’ and ‘rna_seq_transcripts’. The first table has the information about the libraries, including species and experimental treatment. The second one has the data about the digital gene expression of each *Eucalyptus grandis* transcript in each library (fields ‘rpkm’ and ‘read_count’). This table is linked to the ‘transcripts’ table (described in the genomic section) by the field ‘id_transcript’ and to the ‘rna_seq_libraries’ table by the field ‘id_library’.

### Annotation section

The information generated during the annotation pipeline is divided into six tables, one for each biological database used to perform gene annotation (NR, Uniref90, CDD, Swiss-prot and TAIR and GO). All of them contain information about the Blast results, including hit description, e-value and the hit order. All annotation tables refer to the *E. grandis* gene prediction (field ‘id_transcript’) and to the ‘assemblies’ table (described in the EST section) through the field ‘id_assembly’. For the Gene Ontology (GO), we also used four tables (‘gene_ontology’, ‘term’, ‘term2term’, ‘term_definition’) from Gene Ontology Consortium (41) (http://www.geneontology.org/page/lead-database-schema).

### Co-expression section

The information about the co-expression analysis is stored in the table ‘coexpression’. It is linked to the *E. grandis* gene prediction (fields ‘id_transcript1’ and ‘id_transcript2’) keeping the information about the co-expression of two transcripts and the Pearson value of the relationship (field ‘pearson’).

### Web interfaces

We developed a public website (http://www.lge.ibi.unicamp.br/eucalyptusdb) where the users can retrieve all the information of the database and perform new analysis. Searches can be done through sequence ID (Phytozome transcript or assembled sequence), sequence comparisons (using local BLAST) or by a keyword. In addition, it is possible to visualize all expressed transcripts in a RNA-Seq library ordered by its expression level and perform statistical testing for Gene Ontology enrichment analysis using a subset of Phytozome transcript ID as input and *E. grandis* gene prediction as background. In order to guide the users, a detailed manual was constructed containing illustrative examples in each section. Also all datasets described here are available for download in the download area.

### Searching for a keyword

The keyword search is performed in the sequences annotation results (Blast results), where it is possible to construct a complex search by combining operators ‘and’ and ‘or’ into a composite search. When a keyword is inserted for a search, firstly the interface shows the number of occurrences of the keyword in each dataset of the EUCANEXT. With this information, the user can select a dataset to visualize the sequences` IDs related to his search. All IDs are linked to the transcript interface, described in the section ‘Searching for a transcript’.

### Searching for a orthologous genes

To the ortholog search, EUCANEXT uses the gene prediction annotation from *Arabidospis thaliana* and *Populus tricocarpa.* The users can perform keyword search and transcript ID in these organisms to identify the correspondent transcript(s) in *Eucalyptus grandis*. In the case of keyword, EUCANEXT will return all genes with the searched annotation and each correspondent in *Eucalyptus grandis* linked to the transcript interface, described in the section ‘Searching for a transcript’.

### Searching for a gene ontology term

This search is recommended if the user want to find transcripts related to a specific ontology term or related to one ontology function. In the second case, EUCANEXT will search by the inserted keyword in the description of the gene ontology terms. The user can filter the type of the terms (‘biological process’, ‘molecular function’, ‘cellular component’ or all types) and to expand the search to the connections (father and children terms).

### Searching for a transcript

**Figure 3.**
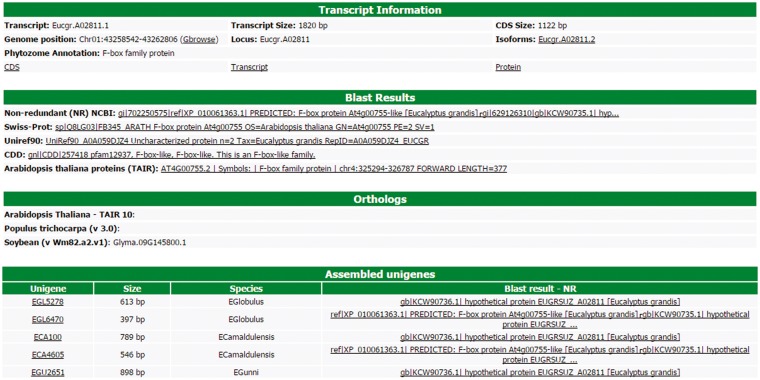
Through this interface, EUCANEXT users can retrieve all information about a transcript, including annotation and digital gene expression in all RNA-Seq libraries (provided in FPKM and read count values).

All information about each transcript is available to the users in a user-friendly interface of the web site (Figure 3). This interface contains six different blocks. The first one, named ‘Transcript information’, presents the basic information about the transcript, such as the name, position in the *Eucalyptus grandis* genome, CDS and transcripts sizes, isoforms, the Phytozome annotation and some links to the FASTA sequences (CDS, transcript and protein). In this block, there is a link to the genome browser (see Gene browser configuration for more information), which shows the exact region of the transcript in the reference genome. The second block (Blast results) presents the annotation of the transcript in the databases, such as NR, Uniref90, Swiss-Prot, Tair, CDD and GO. All Blast results are linked to the original output file allowing the user to view the blast results with a graphical interface. The orthologous relationship between *E. grandis* transcript and *Arabidopsis thaliana*, *Populus trichocarpa* and *Glycine max* are shown in the third block. In the fourth, EUCANEXT shows 10 co-expressed transcripts ordered by the Pearson value (see co-expression analysis section). The fifth block presents the Eucspresso contigs and the unigenes (from the assemblies of all species and from Eucatoul) that were mapped in the same locus of the transcript. Finally, the sixth block shows the RNA-Seq data. Here, the digital expression of the transcript is estimated by two metrics: read count and RPKM (or FPKM) values. The RPKM or FPKM values are used in the case of RNAseq data are single-end or paired-end, respectively. Users can filter the results using a RPKM (or FPKM) cutoff.

### Identifying differentially expressed transcripts

Users can perform differential expression analysis using RNA-Seq samples available at the database. EUCANEXT will construct a read count matrix using samples selected by user and performs a statistical analysis using the DESeq2 package (42) with default parameters. EUCANEXT server will execute the job as background, and send an email to user with a link to download a zip containing seven files: (i) TXT file containing the read count matrix used in the analysis; (ii) CSV output file generated by the DESeq2 package; (iii) PDF file containing the heatmap, PCA and MA plots generated by the DESeq2 package; (iv) Tab-delimited file containing the main results from DESeq2 analysis (transcript name, transcript annotation, log2fold-change and FDR) and RPKM (or FPKM) values from all selected samples; (v) Tab-delimited file (similar item iv) with only the differentially expressed transcripts based on the FDR cut-off selected by the user; (vi) R script file used in the analysis and (vii) Log file generated by R script.

### Gene browser configuration

**Figure 4.**
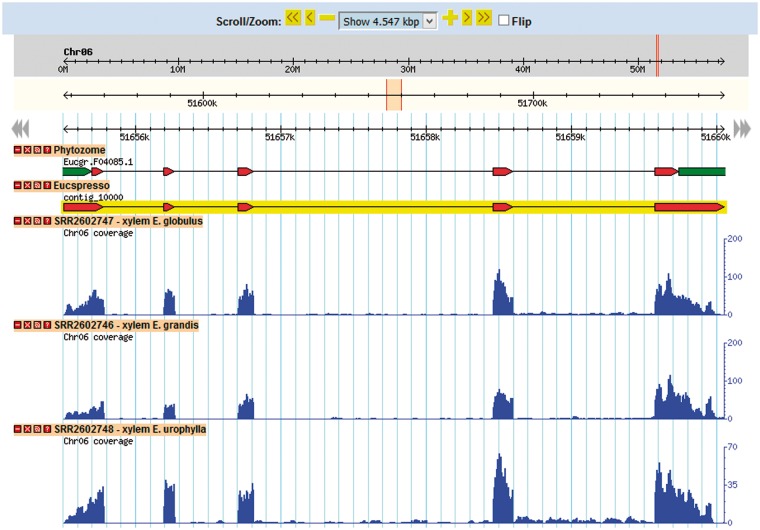
This tool allows for the visualization of a gene in the genomic context. Through its use, it is possible to identify new isoforms of known genes, new genes and SNPs looking for the coverage of mapped reads for the transcript in each library.

The *Eucalyptus grandis* genome assembly and transcript annotation were used as a reference to set the gene browser of EUCANEXT (Figure 4). All assembled ESTs, Eucatool and Eucspresso transcripts were aligned against the *Eucalyptus grandis* genome assembly using the Exonerate software version 2.2.0 (43). The program was configured with the parameters ‘–percent 20 –bestn 1 –geneseed 50 –seedrepeat 10 –quality 80 –refine region’ and set to return all optimal alignments of the transcripts. After a conversion from the exonerate output to gff3, the files were used to configure the sequences position in the reference genome at the genome browser. The RNA-Seq libraries were added in the genome browser using the results of the TopHat alignment of the read against the *Eucalyptus grandis* genome.

### Visualizing top expressed genes

The EUCANEXT platform allows the user to visualize the list of all expressed transcripts and order by its expression values (RPKM or FPKM) in a specific RNA-Seq library. It is very useful information allowing the users to have a broad overview of the expression profiles in a specific tissue or condition.

### Gene ontology enrichment analysis

The statistical testing of enrichment analysis for GO biological process is performed using the hypergeometric distribution (44). The exact hypergeometric distributions were implemented using gamma function (45).

## Discussion

We present EUCANEXT, a database that integrates the *Eucalyptus grandis* genome assembly with transcriptome data from several eucalyptus species, including 165,268 ESTs and 26 Illumina RNA-Seq libraries. The user-friendly web interface was developed to show information about annotation, orthologs relationship and gene expression profiles. The orthologs relationship analysis between *Eucalyptus grandis* and *Arabidopsis thaliana* is particularly useful, considering that there are several *A. thaliana* genes experimentally characterized. Additionally, the *Populus tricocarpha* closely related and well-studied species.

Among the available web-based *Eucalyptus* databases, EUCANEXT is the only one to integrate *Eucalyptus* transcriptomic data from multiple sources and experiments, as only two transcriptome experiments are available in the Eucatoul database (22, 23) and the *Eucalyptus camaldulensis* Genome Database (15) only allows for BLAST searches. The EUCANEXT database was developed with the main purpose of aiding the mining of genes related to important silvicultural properties, such as stress response and productivity that can be obtained by comparison of RNA-Seq data from different species or limiting nitrogen/water conditions. The database provides tools to compare gene expression, allowing for the identification of transcripts expressed in certain species or tissues, and also perform Gene Ontology enrichment analysis using a set of Phytozome transcript ID uploaded by user.

**Figure 5.**
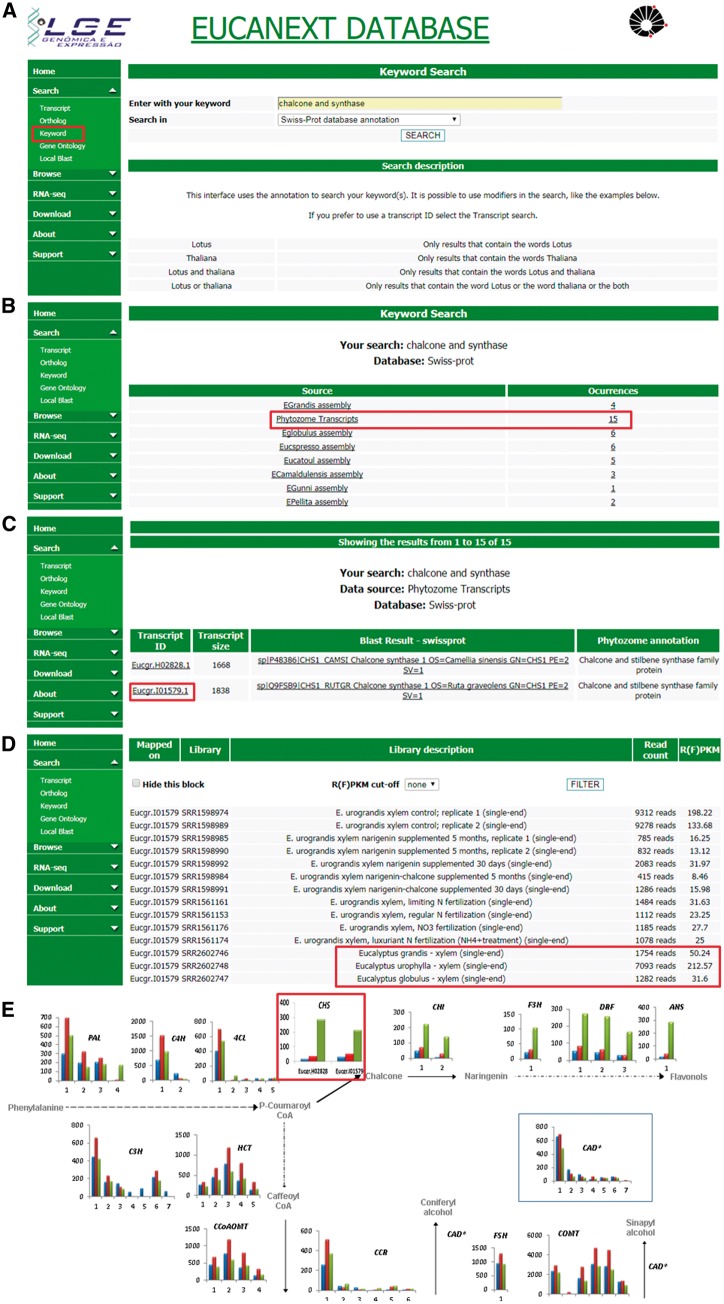
Real case step-by-step example of gene expression analysis into EUCANEXT database. (**A**) Eucanext keyword search of the Chalcone synthase gene (CHS) using ‘chalcone and synthase’ as query. (**B**) CH5 genes from *E. grandis* genome assembly annotated by swiss-prot database. (**C**) EUCANEXT gene expression results for two isoforms of CHS gene identified during RNA-Seq analysis of all libraries including different Eucalyptus species, tissues and conditions. Red square indicates the isoform chosen by sequence length and analysed in (D); (**D**) The selected isoform of CHS gene is at least 4× more expressed in *E. urophylla*, compared to *E. grandis* and *E. globulus*. (**E**) Visual representation of the simplified phenylpropanoid pathway gene expression on three eucalyptus specie constructed based on steps (A–D) and published by our Group (20), where the numbers on the *x*-axis represent different isoforms of each genes and the *y*-axis represents the isoform expression (FPKM values). The colored bars indicate gene expression for different species: *E. globulus* (blue), *E. grandis* (red), *E. urophylla* (green). As described in steps (A–D) and highlighted in this figure by red rectangle, CH5 isoforms from *E. urophylla* showed higher expession levels than *E. globulus* (blue) and *E. grandis* (red).

Figure 5 shows a real case study using the EUCANEXT database (keyword search and gene expression profile tools) for identification of genes related to lignin formation, specifically the phenylpropanoid pathway and their expression profile in three different species known to have variations in the formation of lignin S and G. The differential expression of genes from this pathway, ex: chalcone synthase (CHS), on *E. urophylla*, compared to *E. grandis* and *E. globulus*, published on the work of Salazar and collaborators (20), led us to investigate the effects of flavonoid supplementation into wood formation of eucalyptus species, revealing that the expression of CHS and the sequential steps of its pathway are directly related to lignin content and composition, reducing Klason lignin % and increasing syringyl/guaiacyl (S/G). This work has been published on two separate manuscripts (18, 46) generated a patent deposit, and initiated a larger trial of nutritional management at the International Paper do Brasil fields.

Moreover, to the best of our knowledge, EUCANEXT is the first database to provide all pipelines, facilitating local implementation. All PERL and CGI scripts used to build the database and the web interfaces, respectively, in addition to the SQL schema are available for download in the EUCANEXT website. Therefore, all the tools here described are readily available to be used in other projects that involve sequencing and digital gene expression. Lastly, the database is easily expansible, allowing for the addition of new datasets and can be constantly updated. Research groups interested in housing their RNA-Seq datasets in the EUCANEXT database can make an online submission using the link ‘New submission’ in the menu ‘RNA-Seq’.

## Conclusions

EUCANEXT is a new resource for *Eucalyptus* genomic studies, which integrates digital gene expression with genomic data. The use of the *E.grandis* genome facilitates the incorporation of new data from other sources, such as microRNAs and SNPs, or from RNA-Seq experiments with new experimental conditions. The web-based interface is a valuable tool for the exploration of gene expression data from several *Eucalyptus* species and tissues, facilitating data mining and the identification of transcripts related to properties of interest.

## Availability of data and materials

All data described in this paper are available at the EUCANEXT webpage (http://www.lge.ibi.unicamp.br/eucalyptusdb) and on NCBI (Sanger ESTs) (https://www.ncbi.nlm.nih.gov/nucest/? term=txid3932[Organism: exp]) or SRA (RNA-Seq samples) (http://www.ncbi.nlm.nih.gov/sra) under the SRA IDs: SRR521589, SRR521590, SRR521591, SRR521592, SRR521593, SRR521594, SRR521595, SRR521596, SRR521597, SRR521598, SRR521599, SRR521600, SRR2602746, SRR2602747, SRR2602748, SRR1598974, SRR1598984, SRR1598985, SRR1598989, SRR1598990, SRR1598991, SRR1598992, SRR1561161, SRR1561153, SRR1561174 and SRR1561176.

## Supplementary data

Supplementary data are available at *Database* Online.

## Funding

Center of Computational Engineering and Sciences at Unicamp (FAPESP/CEPID project #2013/08293-7), Project Gene Discovery on Eucalyptus – UNICAMP/FUNCAMP/International Paper do Brasil Ltda (Project numbers 07-P-20180/2007; 07-P-20188/2007; 07-P-30491/2012) and CNPq-Universal grant.

## Author’s contributions

L.C.N. performed the assemblies, the automatic annotation, designed the database and wrote the article. M.M.S., J.L.N. and E.L.O.C. helped in the conception of the database and of the article and reviewed the article. G.A.G.P. and M.F.C. conceived the study, coordinated and helped to draft the article. All authors read and approved the final article.

*Conflict of interest*. None declared.

## Supplementary Material

Supplementary Data
