# Supplementary material for: EUCANEXT: an integrated database for the exploration of genomic and transcriptomic data from Eucalyptus species
Source: Database (Oxford). 2017 Oct 24;2017:bax079. doi: 10.1093/database/bax079 (PMC5737058; doi:10.1093/database/bax079)
Supplement: Supplementary Data [file bax079_additionalfile1.pdf]

EST Sequences						
**Tissue**	***E. camaldulensis***	***E. globulus***	***E. grandis***	***E. gunni***	***E. pellita***	***E. urophylla***
Apex	-	1,336	-	-	-	-
Leaf	14,006	8,737	12,817	-	-	-
Mesophyll	-	-	-	11,303	-	-
Root	13,928	-	60	-	-	-
Seedling	2,873	2,621	21,819	-	-	-
Shoot	5,274	-	-	-	-	-
Stem	21,521	-	-	-	-	-
Xylem	-	16,106	6,920	8,538	8,870	7,431
Others	-	149	959	-	-	-
	57,602	28,949	42,575	19,841	8,870	7,431

Assemblies results						
**Tissue**	***E. camaldulensis***	***E. globulus***	***E. grandis***	***E. gunni***	***E. pellita***	***E. urophylla***
Contigs	8,519	3,868	6,013	3,175	1,404	1,039
Singlets	8,150	6,856	9,230	3,703	2,956	3,008
Total assembled sequences	16,669	10,724	15,243	6,878	4,360	4,047
Annotated (Blastx NR)	11,880	8,663	11,998	4,551	3,551	3,130
Annotated (Blastx Swiss-prot)	9,266	6,934	9,324	3,590	2,819	2,473
Annotated (Blastx TAIR)	11,314	8,417	11,089	4,395	3,471	3,052
